# Daratumumab in the treatment of C3 glomerulopathy with monoclonal gammopathy: a case report and literature review

**DOI:** 10.3389/fmed.2023.1266172

**Published:** 2023-09-01

**Authors:** Pasquale Esposito, Daniela Picciotto, Francesca Costigliolo, Elisa Russo, Lucia Macciò, Giovanna Cenacchi, Antonia Cagnetta, Michele Cea, Roberto M. Lemoli, Francesca Viazzi

**Affiliations:** ^1^Unit of Nephrology, Dialysis, and Transplantation, IRCCS Ospedale Policlinico San Martino, Genoa, Italy; ^2^Department of Internal Medicine and Medical Specialties (DiMI), University of Genoa, Genoa, Italy; ^3^Biotechnology and Methods in Laboratory Medicine, Department of Experimental, Diagnostic and Specialty Medicine (DIMES), University of Bologna, Bologna, Italy; ^4^IRCCS Ospedale Policlinico San Martino, Genoa, Italy; ^5^Clinic of Hematology, Department of Internal Medicine and Medical Specialties (DiMI), University of Genoa, Genoa, Italy

**Keywords:** C3 glomerulopathy, monoclonal gammopathy, MGRS, daratumumab, kidney biopsy, case report

## Abstract

Although rare, C3 glomerulopathy (C3G) is increasingly recognized thanks to the currently available diagnostic skills. C3G is not a single disease but a group of disorders with distinct pathogenesis and progression. Thus, an essential step for its management remains an in-depth characterization of the specific form and the identification of underlying conditions, which may also impact treatment choices as well. Among these entities, an emerging condition is the association of C3G with monoclonal gammopathy, which confers poor outcomes. Overall, diagnosis of C3G remains challenging, and determining the appropriate treatment remains unclear. Conventional immunosuppressive therapy has proven ineffective in such cases, while clone-directed therapies have shown promising results in small interventional studies and case series. Here, we report a case of a patient affected by C3G with monoclonal gammopathy of renal significance who experienced rapid deterioration of kidney function requiring replacement therapy. After the failure of first-line treatment, a switch to the anti-CD38 therapy with daratumumab resulted in the progressive improvement of the patient’s kidney function, leading to the discontinuation of hemodialysis after approximately 10 months. Serial renal biopsies were also performed to study the disease’s evolution in response to the treatment. Based on the description of this single case, we have comprehensively reviewed available studies on daratumumab use in patients with C3G associated with monoclonal gammopathy to provide insights for the design of prospective studies which aim to enhance the management of such poor prognosis disease.

## Introduction

C3 glomerulopathy (C3G) is an extremely rare condition, with an estimated incidence of 1–5 cases per 1,000,000 individuals (1). Rather than a single disease, C3G encompasses a group of kidney disorders characterized by abnormal activation of the alternative pathway of the complement system (2). According to the current guidelines, C3G is defined by the presence of immunofluorescence of C3 deposition in renal tissue at least two orders of magnitude higher than any other immunoreactant (including Ig, C4, C1q, etc.) (3).

Based on ultrastructural findings, C3G is further divided into C3 glomerulonephritis (C3GN), characterized by the presence of immune deposits in the subendothelial space and mesangium of glomeruli, and dense deposit disease (DDD), defined by deposits within the mesangium and highly osmiophilic basement membranes (4).

Complement activation in C3G can be triggered by diverse factors, including circulating autoantibodies against complement components or rare gene variants (5). Nevertheless, C3 deposition can often indicate other types of glomerulonephritis with prominent complement activation beyond C3G, including post-infectious glomerulonephritis or secondary glomerulonephritis associated with systemic diseases (such as lupus nephritis, essential cryoglobulinemia, and others) (6). Therefore, making a differential diagnosis can be challenging, often requiring a comprehensive workup integrating kidney biopsy findings, clinical evaluation, serological assessments, and genetic testing (7). Emerging data suggest the frequent presence of monoclonal component (MC) in the serum of C3G patients, thus supporting a link between lympho/plasma cell disorders and such a rare renal disease (8, 9). Indeed, C3G may be found in patients with overt multiple myeloma (MM), or in those with a MC falling under the definition of monoclonal gammopathy of renal significance (MGRS) (10). Prevalence of C3G-MC among C3G-diagnosed patients ranges from 15 to 40%, and it is often associated with older age and a heightened risk of kidney disease progression (11, 12).

Some cohorts of C3G-affected patients with a MC have shown a high prevalence of C3 nephritic factor and anti-factor H antibodies, suggesting a clonal protein-triggered complement activation (13). However, the exact pathogenic mechanisms underlying this rare clinical condition are still unclear, which leads to unsuccessful therapeutic approaches. Notably, while those patients affected by C3G in the context of MM are treated according to well-defined criteria, the treatment strategy for the cases of C3G-MC in the setting of MGRS is uncertain (14). Administration of immunosuppressive drugs (such as cyclophosphamide, mycophenolate mofetil) and monoclonal antibodies (rituximab) showed scarce efficacy, while experience with complement inhibitor, eculizumab, is limited to a few cases (15). In such a disappointing context, encouraging case reports propose anti-clone targeted therapies, including proteasome inhibitors and anti-CD38 monoclonal antibodies (MoAbs), as potential treatments (16), but a unified therapeutic approach for this high-risk condition remains elusive.

Here, we report a C3G-MC patient who suffered rapid kidney function decline requiring hemodialysis. After a first-line regimen treatment failure, the patient underwent rescue treatment with daratumumab, an anti-CD38 agent, resulting in progressive kidney function improvement. Remarkably, this improvement allowed discontinuation of hemodialysis 10 months after initiation. Throughout the clinical course multiple, kidney biopsies were performed, facilitating a comprehensive analysis of disease progression and treatment effects.

## Case description

In July 2021, a 38-year-old man presented to our Nephrology Division with generalized edema that had started a few weeks before.

His clinical history was unremarkable except for childhood epilepsy. Upon admission, a physical examination revealed anasarca and hypertension. Laboratory investigations revealed mild renal dysfunction with a serum creatinine level of 1.2 mg/dL, corresponding to an estimated glomerular filtration rate (eGFR) of 76 mL/min, with moderate hyperkalemia 5.8 mEq/L. Urinalysis revealed microscopic hematuria at 400/μL and proteinuria of 5 g/24 h with 0.10 g/24 h of Bence Jones at urine protein electrophoresis (UPEP). Serum protein electrophoresis (SPEP) showed low gamma globulin levels of 5 g/L (normal range 8–17 g/L) associated with a monoclonal IgG lambda spike (2.5 g/L). Serum free light chain (FLC) test revealed 34.3 mg/L and 56 mg/L of kappa and lambda, respectively, with a normal ratio of 0.6 (normal range: 0.3–1.56).

Other laboratory tests showed low levels of C3 (0.37 g/L, normal range 0.9–1.8) and C4 (0.08 g/L, normal range 0.1–0.4) levels, Hb 14.9 g/dL, calcium 8.4 mg/dL, total cholesterol 214 mg/dL, NT-proBNP of 1050 ng/L.

Hepatic viral markers, autoimmunity, rheumatic factor, and cryoglobulins were all negative.

**Figure 1:**
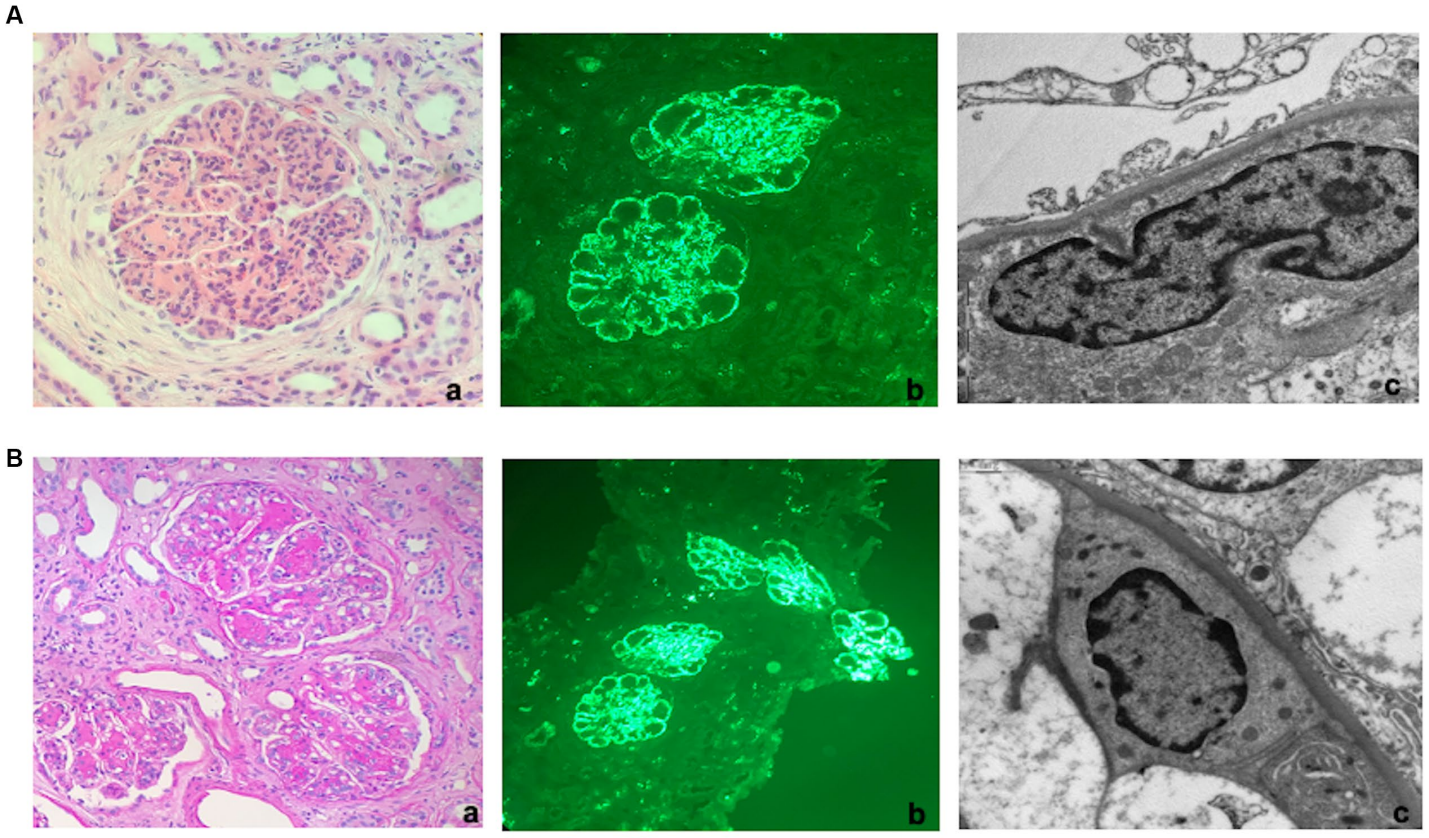
Kidney histology. **(A)** First kidney biopsy (at diagnosis). **(a)** Light microscopy. Lobulated glomerulus with global thickening of the glomerular membranes due to double contours and endocapillary hypercellularity suggestive of membranoproliferative nephritis (TRM 40x). **(b)** Immunofluorescence. Bright granular C3 staining along capillary walls and within mesangial areas. **(c)** Electron Microscopy. Granular powdery electron-dense deposits along the subendothelial space (indicative of a diagnosis of C3 glomerulonephritis). **(B)** Third kidney biopsy (2 months after hemodialysis discontinuation). **(a)** Light microscopy. Glomeruli showed more prominent nodular mesangial matrix expansion than the two previous biopsies but still with lobulation due to hypercellularity, endocapillary proliferation, and double contours (PAS 20X). **(b)** Immunofluorescence. Diffuse granular C3 staining along capillary walls and within mesangial areas. **(c)** Electron Microscopy. Finely granular powdery electron-dense deposits along the subendothelial space.

A percutaneous renal biopsy was then performed. The histological exam revealed a renal cortex with 14 glomeruli, including two globally sclerosed. All the remaining glomeruli were lobulated with severe hypercellularity, both mesangial and endocapillary, and numerous double contours of the glomerular membranes. Immunofluorescence revealed diffuse bright C3 staining (3+) without other positive immune reactants and masked deposits on pronase-digested tissue. Electron microscopy confirmed the presence of subendothelial electron-dense deposits, indicative of a diagnosis of C3GN (Figure 1A).

To further complete the diagnosis workup, comprehensive alternative complement system genetic (*C3, CD46, THBD, DGKE, CFHR1, CFHR3 e CFHR5*) and serological evaluations (C3Nef, anti-H antibodies, soluble C5b9 levels) were performed. All these tests were negative, except for slightly elevated soluble C5b9 levels (309 ng/mL, normal range 140–280 ng/mL). Due to the presence of a monoclonal component at SPEP, a bone marrow biopsy was also done: no tumor infiltration was detected, but cytofluorometric analysis revealed a population of CD138 positive plasma cells with irregular phenotype, accounting for 0.1% of cells, together with polytypic B-lymphocytes and plasma cells. FISH analyses on selected CD138-positive cells did not detect any chromosomal aberration. Finally, spine MRI and positron emission tomography-computed tomography showed no evidence of abnormalities. Overall, the data allowed to diagnose C3G-MC in the context of MGRS. Thus, in November 2021, the patient started an anti-MM-like treatment: the CyBorD schedule (Velcade 1.5 mg/mq, Cyclophosphamide 300 mg/m^2^, and dexamethasone 40 mg given on days 1–8–15–22). At that time, creatinine level was 2 mg/dL, associated with nephrotic range proteinuria 9.4 g/die; SPEP and UPEP analyses were like those performed at diagnosis. Unfortunately, treatment was discontinued early (at the second cycle) due to grade 3 neuropathy, resulting in seizures, hypertension, headache, lethargy, confusion, and blindness.

After treatment withdrawal, the patient showed a progressive worsening of renal function. Indeed, serum creatinine ranged from 2.7 to 3.1 mg/dL in November and December, respectively. In February 2022, the patient was admitted to the emergency department for dyspnea and edema. Laboratory investigations showed serum creatinine of 7.7 mg/dL, eGFR 8 mL/min with hyperkalemia 5.5 mEq/L, low C3 0.6 g/L, normal C4, severe nephrotic proteinuria (7.6 g/24 h) and microscopic hematuria (500/μL). SPEP confirmed hypogammaglobulin levels (6 g/L) together with monoclonal IgG lambda spike (3.4 g/L); serum FLC ratio was still normal (0.61). Due to the severity of the illness, intermittent hemodialysis (HD) was initiated, and a second kidney biopsy was performed to assess kidney damage severity.

The second kidney biopsy confirmed the previous findings, with the addition of some extracapillary cellular proliferation forming crescents. The tubulo-interstitium showed mostly acute findings, with diffuse simplification of the tubular epithelium and severe interstitial lymphocytic inflammation: the overall degree of tubular atrophy and interstitial scarring was mild. Considering the persistence of active lesions with only mild chronicity, in March 2022, the patient underwent a second-line treatment with Methylprednisolone and cyclophosphamide at the dose of 12 mg/kg, according to the KDIGO guidelines for rapidly progressive glomerulonephritis (17).

The first infusion was well-tolerated, and the patient continued therapy with cyclophosphamide and maintenance thrice-weekly HD.

Approximately 1 month later, with the prospect of prescribing a second-line clone-directed therapy, hematologists opted for a second bone marrow biopsy. This examination revealed the presence of monoclonal plasma cells ranging from 5 to 10% of cells with normal hematopoietic cells without significant maturation delay. Remarkably, a reactive infiltrate of lymphocytes was observed. Thus, considering the histological evidence of a plasma cellular clone infiltration, the treatment was shifted to an anti-CD38 MoAb-based strategy. So, in May 2022 patient started daratumumab-lenalidomide-dexamethasone (D-RD, 4-week cycle regimen). The treatment was well-tolerated, yielding a progressive recovery of renal function, increased diuresis, reduced intradialytic weight gain, and improved pre-dialysis examination results. During this period, C3 levels remained consistently low, while C4 levels maintained normal.

Regrettably, in November 2022, at the 7th cycle of therapy, pneumonia occurred, and treatment was interrupted. Afterward, therapy was resumed, but it was switched to single agent daratumumab as maintenance. In the following few months, HD frequency progressively reduced till December 2022, when it was interrupted (10 months after HD initiation and 7 months after the daratumumab starting date). At that time, the patient was clinically euvolemic, with a serum creatinine level of 3.1 mg/dL and proteinuria of 4 g/24 h. On February 2023, while the patient’s renal function was stable and the monoclonal component reduced to 1.1 from 3.5 g/L, a third kidney biopsy was performed (Figure 1B).

Similar to the previous examinations, this biopsy demonstrated that the glomeruli retained a lobulated appearance with a membranoproliferative pattern, albeit with less hypercellularity and increased mesangial matrix deposition, presenting as a vague nodular pattern. The degree of inflammation and acute tubular injury had diminished, though the overall level of interstitial scarring remained mild. Immunofluorescence analysis once again revealed intense bright staining for C3 as the sole immunoreactant. Electron microscopy confirmed the presence of powdery electron-dense deposits along the glomerular membranes, though these deposits were more segmental compared to the prior two biopsies.

**Figure 2:**
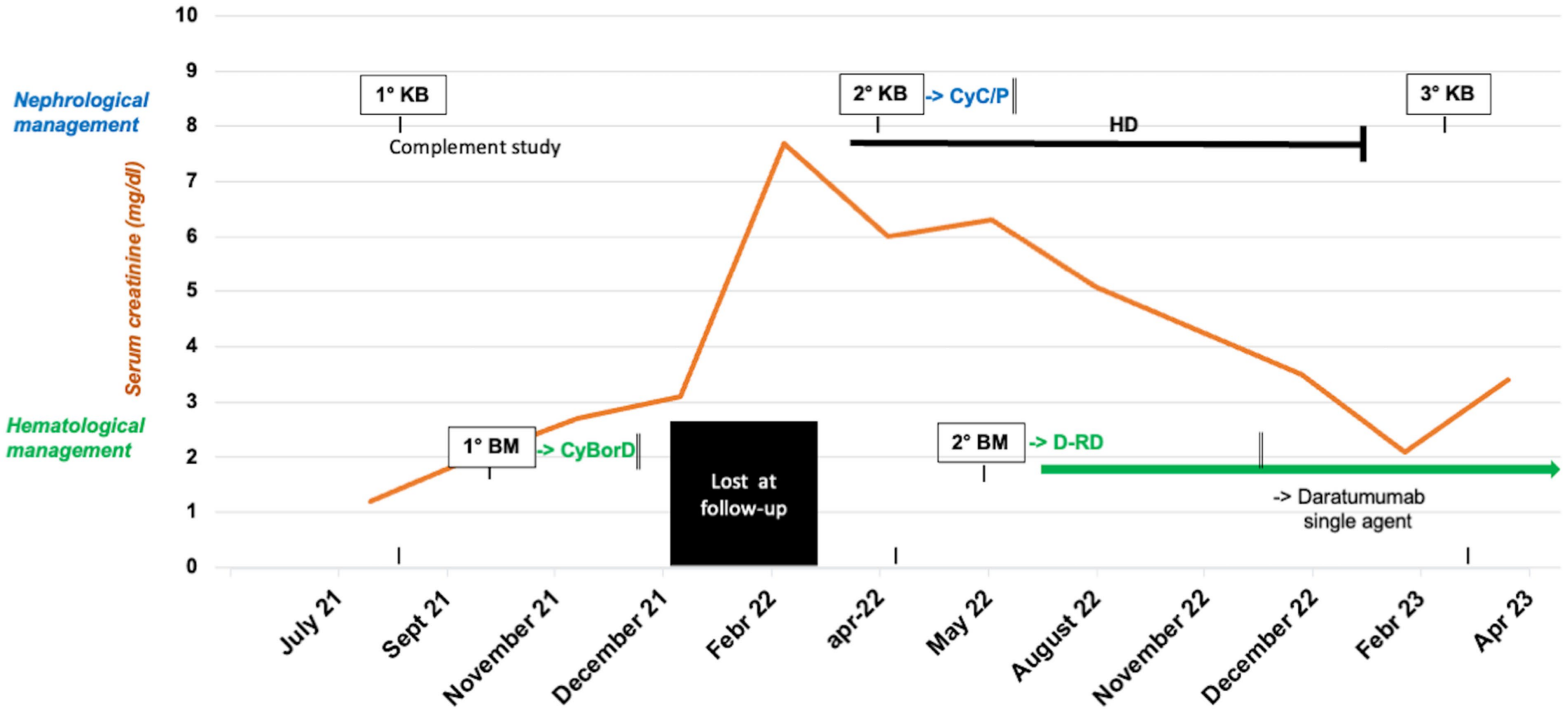
Clinical course, timeline of histological exams and response to the treatments of the index case. kidney biopsy (KB), bone marrow biopsy (BM), cyclophosphamide (Cyc), prednisone (P), bortezomib/cyclophosphamide/dexamethasone (CyBorD), hemodialysis (HD), daratumumab-lenalidomide-dexamethasone (D-RD).

Based on a comprehensive assessment of the patient’s clinical and histological features of active disease, in consultation with the hematologist, we decided to continue the treatment. By April 2023, after the completion of the 14th treatment cycle, the patient’s renal function had stabilized, with a creatinine level of 3.2 mg/dL, proteinuria of 2.5 g/24 h, and slightly low C3 levels (0.82 g/L) and normal C4 (0.28 g/L). The monoclonal component remained at 1.5 g/L. The patient continued maintenance therapy with monthly administration of daratumumab as a single agent (Figure 2).

## Discussion

We present a unique case of a patient with a history of rapidly progressive kidney failure due to C3G-MC who showed an impressive response to anti-CD38 monoclonal antibody treatment.

C3G-MC is a recently identified clinical entity with an unclear classification and a challenging diagnosis (8). In most cases, it falls within the spectrum of MGRS, which includes other complement activation-related diseases, like cryoglobulins and proliferative glomerulonephritis with monoclonal Ig deposits (PGNMID) (17). In these conditions, the underlying causes of complement dysregulation are often difficult to be defined (18). Genetic tests are usually negative and complement-directed autoantibodies are detected in only about 50% of the cases (7).

Identifying monoclonal components and clones is challenging in the absence of overt MM (19). In our patient, a small circulating monoclonal component was found initially, with monotypic plasma cells identified later. Whether this finding results from disease evolution or test sensitivity remains unclear. Importantly, it is now certain that untreated C3G-MC, like other MGRS forms, leads to poor renal outcomes and high recurrence rates post-transplantation (9, 20). However, there is still no consensus on the correct treatment options for these patients (21). In the past years, supportive or immunosuppressive therapies have led to high end-stage kidney disease (ESKD) rates (22).

More recently, clone-directed treatments derived from those used for MM or lymphoma have shown improved renal outcomes (23). The most relevant results from clone-directed therapies in C3G-MC are those obtained with bortezomib (24). Unfortunately, side effects often occur after this treatment, thus limiting their use in patients (25). For example, our patient experienced early neurological toxicity to bortezomib, leading to a complete withdrawal of the treatment. Recently, daratumumab, a CD38-targeting fully human monoclonal antibody, has shown promising results in the treatment of plasma cell dyscrasias (26). The binding of daratumumab with CD38 induces cell death through different Fc-mediated mechanisms: apoptosis, phagocytosis, and antibody- as well as complement-dependent cytotoxicity (27). Daratumumab causes an increase in the number of T cells in the bone marrow and blood, which results in a reinforcement of the immune response against malignant clonal cells (28). Based on the impressive results observed in several randomized phase III clinical trials (29–34), daratumumab is currently approved for the treatment of plasma cell tumors at different stages of the disease. This treatment has shown a good safety profile with the most common adverse events represented by infusion-related reactions, hematological toxicity, and an augmented risk of respiratory infections (35). Remarkably, daratumumab-based triplet and quadruplet combinations were safely administered to MM patients with severe renal impairment (eGFR <30 mL/min or on dialysis), where it may reduce dialysis frequency or lead to dialysis withdrawal (36).

As a result, emerging data report the efficacy of anti-CD38 monoclonal antibodies for C3G-MC and other forms of MGRS. Qin et al. reported the case of a patient with kappa-light chain proximal tubulopathy and crystal-storing histiocytosis who, after the combined treatment with daratumumab and bortezomib, achieved a very good partial response and stabilization of the renal disease (37). Coltoff et al. described two cases of C3G associated with an IgG kappa MC who did not respond to the treatment with CyBorD and instead presented an improvement in renal function after the switch to daratumumab-based regimens (38). In a retrospective analysis by Kastris et al. 25 patients with various forms of MGRS, including C3G and PGNMID, were treated with daratumumab-based regimens (39). Importantly, 12 of these patients were previously untreated, and daratumumab was the first-line therapy, either alone or in combination with other agents. Overall, the treatment was well-tolerated, with better renal and hematological response rates observed when daratumumab was combined with other agents and used upfront compared to daratumumab used as a single agent and as a second-line therapy. Presently, only one prospective clinical trial has evaluated the safety and efficacy of daratumumab alone in 10 patients with PGNMID, 7 of whom were previously untreated (40). Daratumumab was administered intravenously at a dose of 16 mg/kg once a week for 8 weeks, followed by 8 infusions every 2 weeks, with a median follow-up of 12 months. The primary safety analysis revealed five severe adverse events, two major infections, and no cases of grade 3 or 4 anemia, leukopenia, or thrombocytopenia. In terms of efficacy, proteinuria significantly decreased, and kidney function remained stable at the end of the follow-up period.

Our patient’s case is distinct due to severe kidney injury and remarkable renal response to daratumumab, started after failed standard treatment and delayed monoclonal plasma cell evidence.

Nonetheless, daratumumab led to the partial recovery of kidney function.

Thus, our case suggests considering daratumumab as a treatment option for C3G-MC patients, regardless of disease progression.

An additional feature deriving from our case report is the histological course analysis of the disease achieved with serial renal biopsies. In particular, the evidence of a low grade of chronicity resulted crucial to consider new therapeutic options, re-evaluating the hematological profile, and leading us to start a daratumumab-based strategy (41). Furthermore, the third biopsy showed a reduction in electrodense deposits, but the stability of the other findings, such as membranoproliferative pattern and C3 glomerular deposition, thus suggesting that clinical recovery induced by daratumumab did not result in a complete histological resolution. Notably, these data deserve further evaluation over time and raise questions regarding the long-term management of daratumumab-based approaches.

Finally, C3 levels were constantly low during the disease development, while C4 rapidly normalized during the treatment. Therefore, we did not observe a clear correlation between circulating complement levels and clinical course, as well as with the response to therapy.

Overall, while daratumumab treatment seems promising for C3G-MC, several questions, including the superiority of combined regimens over monotherapy and the optimal treatment duration, remain still open. Waiting for more answers, our case report suggests daratumumab treatment as a valuable therapeutic option for patients with C3G associated with MC, at least in the presence of measurable clonal CD38+ plasma cells. Meticulous follow-up, including clinical, laboratory, and histological investigations, is crucial for maximizing treatment benefits.

Prospective multicenter studies with serial kidney biopsies are needed for clarification.

## Data availability statement

The raw data supporting the conclusions of this article will be made available by the authors, without undue reservation.

## Ethics statement

Written informed consent was obtained from the individual(s) for the publication of any potentially identifiable images or data included in this article.

## Author contributions

PE: Conceptualization, Writing – original draft. DP: Writing – original draft. FC: Data curation, Writing – original draft. ER: Conceptualization, Methodology, Writing – original draft. LM: Data curation, Writing – original draft. GC: Data curation, Writing – original draft. AC: Investigation, Writing – original draft. MC: Data curation, Validation, Writing – review & editing. RL: Writing – review & editing. FV: Writing – review & editing.

## Funding

The author(s) declare that no financial support was received for the research, authorship, and/or publication of this article.

## Conflict of interest

The authors declare that the research was conducted in the absence of any commercial or financial relationships that could be construed as a potential conflict of interest.

The author(s) declared that they were an editorial board member of Frontiers, at the time of submission. This had no impact on the peer review process and the final decision.

## Publisher’s note

All claims expressed in this article are solely those of the authors and do not necessarily represent those of their affiliated organizations, or those of the publisher, the editors and the reviewers. Any product that may be evaluated in this article, or claim that may be made by its manufacturer, is not guaranteed or endorsed by the publisher.
